# *Caenorhabditis* Intervention Testing Program: the creatine analog β-guanidinopropionic acid does not extend lifespan in nematodes

**DOI:** 10.17912/micropub.biology.000207

**Published:** 2020-01-02

**Authors:** Anna L. Coleman-Hulbert, Erik Johnson, Christine A. Sedore, Stephen A. Banse, Max Guo, Monica Driscoll, Gordon J. Lithgow, Patrick C. Phillips

**Affiliations:** 1 Institute of Ecology and Evolution, University of Oregon, Eugene, Oregon 97403, USA; 2 Division of Aging Biology, National Institute on Aging, Bethesda, Maryland 20892, USA; 3 Department of Molecular Biology and Biochemistry, Rutgers University, Piscataway, New Jersey 08854, USA; 4 The Buck Institute for Research on Aging, Novato, California 94945, USA

**Figure 1: Longevity under adult β-guanidinopropionic acid exposure:**
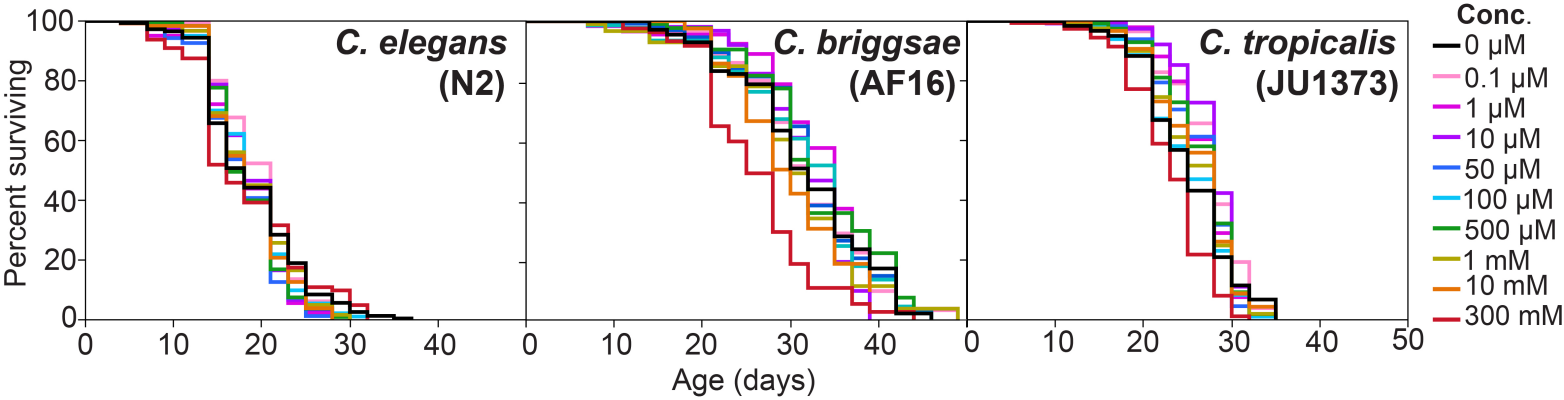
Survival curves for *C. elegans* strain N2, *C. briggsae* strain AF16, and *C. tropicalis* strain JU1373 exposed to β-guanidinopropionic acid at various concentrations starting on the first day of adulthood. Only the lifespan of AF16 at 300 mM (mean=26.2 days, *p*=0.0123) differed significantly from the control (mean=31.5 days for AF16; control means for N2=18.8 days and JU1373=25.1 days; statistical comparisons were made with a Cox proportional hazards mixed-model using the coxme v.2.2-5 package in R (Therneau 2012; Lucanic *et al.* 2017)).

## Description

The *Caenorhabditis* Intervention Testing Program (CITP) is a multi-institutional, National Institutes of Aging (NIA)-funded consortium charged with identifying chemical compounds that robustly extend lifespan in a genetically diverse panel of *Caenorhabditis* strains. Compounds are prioritized for screening if they are highly ranked via computational prediction for lifespan or healthspan effects (Coleman-Hulbert *et al.* 2019), if they are predicted to engage known lifespan regulating pathways, or if they have previously been reported as extending lifespan or healthspan in model systems (Lucanic *et al.* 2017). β-guanidinopropionic acid (β-GPA) is a creatine analog (Shields and Whitehair 1973), commonly used as a dietary supplement, and has been shown to extend lifespan in *Drosophila* under stress via 5′ AMP-activated protein kinase (AMPK) activity (Yang *et al.* 2015). The AMPK pathway is conserved in nematodes and humans (Apfeld *et al.* 2004) and is involved in multiple pathways affecting stress response and metabolism (Wang *et al.* 2012).

We assayed lifespan in response to β-GPA exposure in three *Caenorhabditis* species using our previously published workflow (Lucanic *et al.* 2017). In brief, worms were age-synchronized by timed egg-lays on standard 60 mm diameter Nematode Growth Media (NGM) plates and transferred at a density of 50 individuals per 35 mm treated plate in triplicate when they reached adulthood (for control plates, there were six replicates of 50 animals each). β-GPA (Sigma-Aldrich) was dissolved in water and diluted appropriately such that addition of 125 µl of solution to 35 mm diameter plates containing NGM with lawns of *E. coli* OP50-1 and 51 µm FUdR would generate the following final β-GPA concentrations: 0.1 µM, 1 µM, 10 µM, 50 µM, 100 µM, 500 µM, 1 mM, 10 mM and 300 mM. Worms were maintained at 20 °C and moved to fresh plates on the first, second, and fourth (*C. tropicalis*) or fifth (*C. elegans* and *C. briggsae*) day of adulthood, then once weekly afterward. Due to our previous experience with compound interventions that potentially alter bacterial viability through transient pH changes upon plate treatment (Banse *et al.* 2019), we investigated the effects of β-GPA on our assay plates. At the above listed concentrations, β-GPA-treated plates had a pH of 6.5 and the bacterial lawns survived treatment when tested by replica plating. Thrice weekly, we observed animals for spontaneous movement or movement after gentle perturbation with a 0.2 mm diameter platinum wire. Death was scored as a lack of movement.

Our results indicate that β-GPA does not extend lifespan in the three nematode species at the concentrations tested here; in fact, in only one instance was an effect detected and, in that case, the compound reduced lifespan (Fig. 1). This conclusion is based on one biological replicate per concentration and, as such, could be considered preliminary. While interventions may be ineffective due to a range of causes, including permeability barriers, compound stability *in* *vivo*, and metabolism by the bacterial food source, we believe that the lack of response in this study was due to a lack of physiological relevance in *C. elegans*. In *Drosophila* and mammalian models, β-GPA reduces the level of intracellular phosphocreatine that can be used by creatine kinases to regenerate ATP (Oudman *et al.* 2013; Yang *et al.* 2015) resulting in a decreased cellular ATP/AMP ratio, which activates AMPK and ultimately increases lifespan and stress-resistance. Our *a priori* expectation for lifespan extension in *C. elegans* was built on the observations that: (1) creatine is reported as detectible in *C. elegans* (Atherton *et al.* 2008; Jones *et al.* 2012; Wan *et al.* 2017); (2) *C. elegans* has a creatine-like kinase, ARGK-1, whose activity modulates AMPK signaling (McQuary *et al.* 2016); (3) modulation of AMPK (Apfeld *et al.* 2004; Greer *et al.* 2007) and ARGK-1 (McQuary *et al.* 2016) in *C. elegans* can affect lifespan and stress resistance; and (4) regulation of lifespan by insulin signaling is partially dependent on AMPK signaling in *C. elegans* (Tullet *et al.* 2014). As such, we were surprised to find no changes in lifespan upon treatment with a creatine analog. One possible explanation is that, despite the similarity between ARGK-1 and mammalian creatine kinase, the enzymes’ substrates differ. Biochemical analysis suggests that ARGK-1 uses arginine instead of creatine as a substrate to recharge ADP (Fraga *et al.* 2015). Additionally, it has been postulated that the biochemical characterization of *C. elegans* metabolites may have misidentified creatine, and that creatine is not relevant to *C. elegans* physiology (Witting *et al.* 2018). Given these caveats, it may not be surprising that β-GPA does not alter *Caenorhabditis* lifespan.
